# The decline in synaptic GluN2B and rise in inhibitory neurotransmission determine the end of a critical period

**DOI:** 10.1038/srep34196

**Published:** 2016-09-28

**Authors:** Noriko Isoo, Takae Ohno, Mutsumi Isowaki, Satoshi Fukuda, Naoyuki Murabe, Hiroaki Mizukami, Keiya Ozawa, Masayoshi Mishina, Masaki Sakurai

**Affiliations:** 1 Department of Physiology, Teikyo University School of Medicine, 2-11-1 Kaga, Itabashi-ku, Tokyo 173-8605, Japan; 2 Division of Genetic Therapeutics, Center for Molecular Medicine, Jichi Medical University, 3311-1 Yakushiji, Shimono, Tochigi 329-0498, Japan; 3 Division of Genetic Therapeutics, the Institute of Medical Science, the University of Tokyo, 4-6-1 Shirokanedai, Minato-ku, Tokyo 108-8639, Japan; 4 Department of Molecular Neurobiology & Pharmacology, Graduate School of Medicine, University of Tokyo, 7-3-1 Hongo, Bunkyo-ku, Tokyo 113-8655, Japan; 5 Brain Science Laboratory, The Research Organization of Science and Technology, Ritsumeikan University, 1-1-1 Nojihigashi, Kusatsu, Shiga 525-8577, Japan

## Abstract

Neuronal plasticity is especially active in the young, during short windows of time termed critical periods, and loss of a critical period leads to functional limitations in the adults. The mechanism that governs the length of critical periods remains unknown. Here we show that levels of the NMDA receptor GluN2B subunit, which functions as a Ca^2+^ channel, declines in spinal cord synapses toward the end of the critical period for activity-dependent corticospinal synapse elimination. This period could be prolonged by blocking the decline of GluN2B, and after its termination the critical period could be reopened through upregulation of GluN2B. It is known that inhibitory neural activity increases with development in the CNS including the spinal cord. Suppression of the increasing inhibitory activity using low-dose strychnine also prolonged this critical period. During the strychnine-widened time window, Ca^2+^ influx through GluN2B channels returned to a level comparable to that seen during the critical period, though the level of GluN2B was slightly reduced. These findings indicate that loss of GluN2B subunits and the associated reduction in Ca^2+^ influx determines the end of the critical period in our *in vitro* CS system.

So-called “critical periods” are specific early postnatal time windows during which levels of activity-dependent neuronal plasticity are heightened. The mechanism by which neuronal activity during a critical period irreversibly alters brain development is a central issue in neuroscience that has been intensively studied using various model systems of the central nervous system, including models of the visual^1^,^2^,^3^,^4^ and somatosensory^5^,^6^,^7^ systems. In our *in vitro* model of rodent corticospinal (CS) projections^8^, CS synapses are formed over the entire spinal cord, but synapses are eliminated as their axons regress from the ventral side of the cord in an activity-dependent manner^9^,^10^. Similar reorganization with a similar time course has been observed *in vivo*^11^,^12^,^13^,^14^. This elimination of CS synapses *in vitro* requires NMDA receptor (NMDAR) activation^9^, and the time window during which it occurs (6–11 days *in vitro* (DIV)) can be considered a critical period. When, for example, the NMDA antagonist D-(-)-2-amino-5- phosphonovaleric acid (APV) is added to the medium to block elimination during the critical period is then removed, the ventral CS synapses are no longer eliminated^15^. This system thus provides a basic model of a critical period.

NMDARs are heterotetramers composed of two GluN1 and two GluN2 subunits. Among the known GluN2 isoforms, GluN2B (2B) is thought to be specifically involved in certain types of developmental plasticity, as it exhibits slower kinetics and mediates higher Ca^2+^ influx than GluN2A (2A)^16^. Moreover, the NMDAR subunit composition shifts from 2B to 2A during development^17^,^18^,^19^,^20^, increasing the likelihood that the decline in 2B is a determinant in the termination of the critical period. Manipulation of the balance of excitatory-inhibitory neural activity has also been shown to alter the onset and closure of the critical period in the developing visual cortex^21^. Given that the numbers of glycine receptors in the ventral spinal cord increase progressively toward adulthood^22^,^23^,^24^, it is possible that the development of inhibitory activity is also involved in closing critical periods. Here we tested these two possibilities using our model system of developmental CS synapse elimination.

## Results

### Decline in 2B-containing NMDARs across the end of the critical period

**Figure 1:**
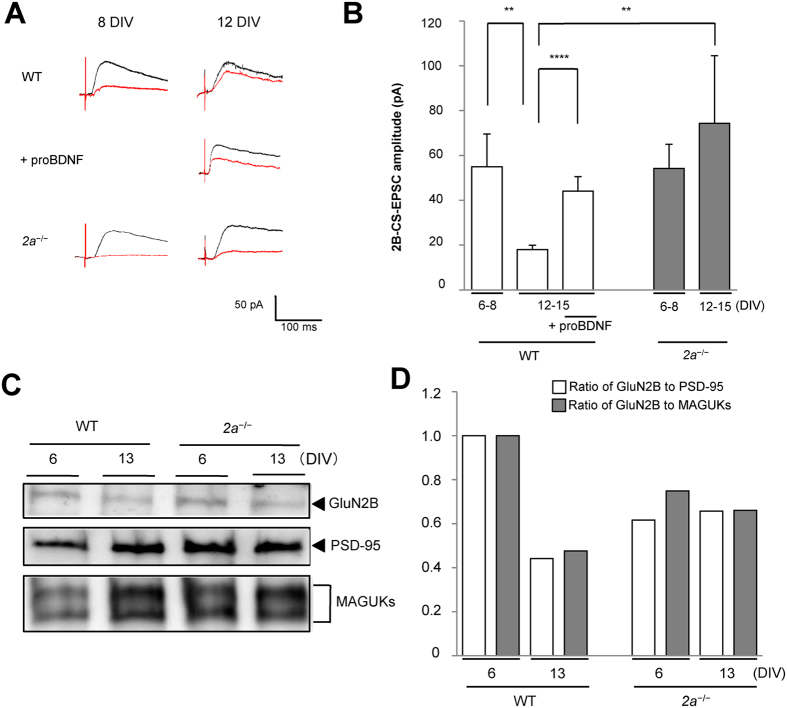
Decline in 2B-containing NMDAR across the end of the critical period. (**A**) Developmental alterations of the total NMDA component of CS-EPSCs in the spinal cord. Averaged CS-EPSC traces recorded after application of NBQX to WT (*upper*) or proBDNF-treated (*middle*) and *2a*^−/−^ (*lower*) mice before (*black*) or after (*red*) Ro25-6981 treatment on 12 DIV. Calibration: 50 pA, 100ms. (**B**) Averaged 2B-CS-EPSCs in WT (55.0 ± 14.6 pA, 6–8 DIV, n = 21, Ns = 16, Nm = 32; 18.0 ± 1.99 pA, 12–15 DIV, n = 18, Ns = 10, Nm = 20), proBDNF-treated (44.1 ± 6.45 pA, 12–15 DIV, n = 13, Ns = 8, Nm = 16) and *2a*^−/−^ mice (54.2 ± 10.8 pA, 6–8 DIV, n = 7, Ns = 7, Nm = 14; 74.4 ± 30.1 pA, 12–15 DIV, n = 7, Ns = 6, Nm = 12). (**C**) Developmental alterations of synaptic 2B in WT or *2a*^−/−^ mice. Immunoblot analysis of synaptic expression of 2B (2B in SPMs), PSD-95 (in SPMs) and MAGUKs (in SPMs) in WT and *2a*^−/−^ mice. Full-length blots are presented in Supplementary Fig. 7S. **(D)** Ratio of 2B to PSD-95 or MAGUKs in SPMs: To quantitate synaptic 2B, the intensity of 2B in SPMs was normalized to that of PSD-95 or MAGUK in SPMs in WT or *2a*^−/−^ spinal cords (Ratio of 2B to PSD-95: 1.00, 6 DIV in WT mice; 0.44, 13 DIV in WT mice; 0.62, 6 DIV in *2a*^−/−^mice; 0.66, 13 DIV in *2a*^−/−^mice. Ratio of 2B to MAGUKs: 1.00, 6 DIV in WT mice; 0.48, 13 DIV in WT mice; 0.75, 6 DIV in *2a*^−/−^ mice; 0.66, 13 DIV in *2a*^−/−^ mice. Each sample represents an SPM extract prepared from 20 co-cultures from 4 mice). Data are represented as the mean ± standard error of the mean. Asterisks indicate statistical significance (Student *t* test). *p < 0.05, **p < 0.01, ***p < 0.005, and ****p < 0.001. CS-EPSC, corticospinal-excitatory postsynaptic current; DIV, days *in vitro*; WT, wild type; MAGUK, membrane-associated guanylate kinase; SPM, synaptic plasma membrane; n, the number of recordings; Ns, the number of slice cultures; Nm, the number of mice.

To measure the 2B component of NMDA currents (2B-CS-EPSCs), we stimulated CS axons in deep layers of the cortex and recorded CS-excitatory postsynaptic currents (CS-EPSCs) from neurons in the spinal cord^8^,^10^,^25^. The 2B component of the currents was measured as the portion of the total NMDA component of CS-EPSCs that was sensitive to the 2B-blocker Ro25-6981 (Fig. 1A). The average amplitudes of 2B-CS-EPSCs after the critical period (12–15 DIV) in wild-type (WT) mice were approximately one-third of those during the critical period (6–8 DIV). By contrast, for *Grin2a*^−/−^ (*2a*^−/−^) mice, in which 2B could not be replaced by 2A, the amplitudes of 2B-CS-EPSCs recorded during the critical period did not differ from those recorded after it (Fig. 1B). In addition, the levels of 2B within synaptic plasma membranes (SPMs) after the critical period (13 DIV) were less than 50% of those during the critical period (6 DIV) in WT mice, but were unchanged in *2a*^−/−^ mice (Fig. 1C,D). These findings indicate that synaptic 2B levels in the spinal cord decline greatly across the end of the critical period.

### Prolongation of the critical period in *2a* ^−/−^ Mice

**Figure 2:**
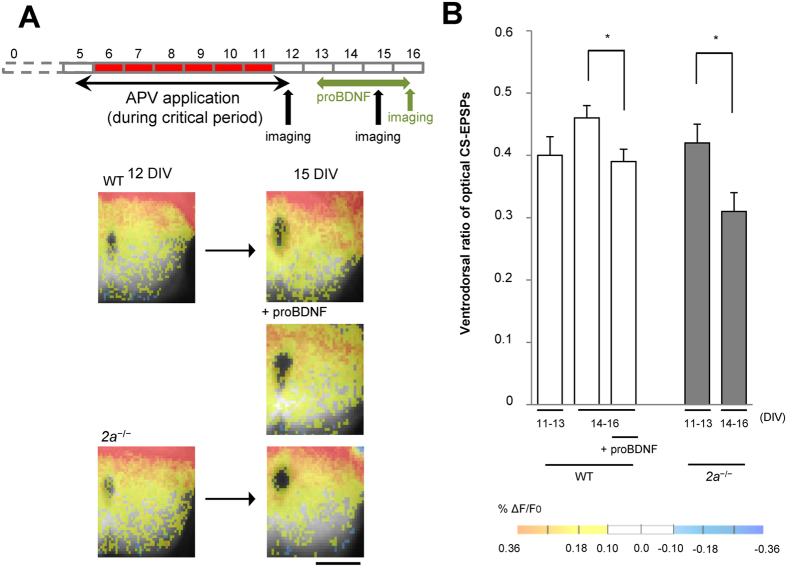
CS synapses in the ventral spinal cord were eliminated upon increasing synaptic 2B. (**A**) Reduction of CS synaptic activity in the ventral side after the critical period in *2a*^−/−^ mice. Spatial distribution of CS synapses determined using optical CS-EPSPs in WT, proBDNF-treated or *2a*^−/−^ mice at 11–13 or 14–16 DIV. Scale bar = 250 μm. (**B**) Ventrodorsal ratios of optical CS-EPSPs in the indicated culture pairs from 11–13 DIV (0.40 ± 0.03 in WT mice, n = 12, Ns = 12, Nm = 6; 0.42 ± 0.03 in *2a*^−/−^ mice, n = 6, Ns = 6, Nm = 6) or from 14–16 DIV (0.46 ± 0.02 in WT mice, n = 17, Ns = 17, Nm = 8; 0.39 ± 0.02 in proBDNF-treated mice, n = 4, Ns = 4, Nm = 4; 0.31 ± 0.03 in *2a*^−/−^ mice, n = 7, Ns = 7, Nm = 6).

**Figure 3:**
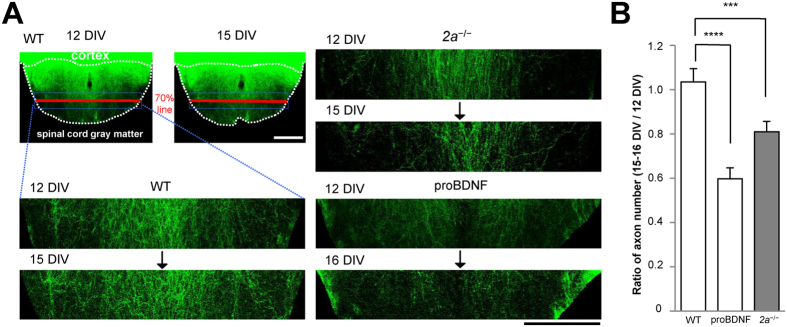
CS axons in the ventral spinal cord regressed upon increasing synaptic 2B. (**A**) Images of CS axons labeled with ChR2-EYFP in WT (*left*), proBDNF-treated and *2a*^−/−^ spinal explants (*right*). The first image was taken at 12 DIV and the second at 15 or 16 DIV. Enumeration of CS axons on the ventral side that crossed the 70% line (*red bold line*) from the dorsal to the ventral edge of spinal gray matter. Scale bar = 250 µm. (**B**) Ratios of CS axons on the ventral side at 15 or 16 DIV to that at 12 DIV (15 DIV/12 DIV: 1.13 ± 0.06 in WT mice, n = 9, Ns = 9, Nm = 4; 16 DIV/12 DIV: 0.60 ± 0.05 in proBDNF-treated mice, n = 11, Ns = 11, Nm = 6; 0.81 ± 0.05 in *2a*^−/−^ mice, n = 11, Ns = 11, Nm = 8) (*right*).

We next optogenetically stimulated CS axons using EYFP-tagged channelrhodopsin 2 (ChR2-EYFP) after infecting cortical slices with a recombinant adeno-associated viral (AAV) vector encoding the protein. We then recorded the spatial distribution of CS synapses through optical imaging with the voltage-sensitive dye RH1691 (optical CS-EPSPs)^10^ (Fig. 2 and Supplementary Fig. 1S). When synapse elimination during the critical period was inhibited by applying APV, the CS synaptic responses on the ventral side of WT spinal cords were unchanged throughout the observation period^15^ (Fig. 2A,B). However, the same manipulation decreased the numbers of CS synapses on the ventral side in *2a*^−/−^ mice at 14–16 DIV, when the critical period would have been over in WT mice (Fig. 2A,B). When we counted the number of CS axons on the ventral side that crossed a 70% line drawn from the dorsal to the ventral edge of the spinal gray matter, we found that the number of CS axons on the ventral side had decreased by more than 20% after the critical period (15 DIV) in *2a*^−/−^ mice, but not in WT mice (Fig. 3). These results indicate that the critical period was prolonged by inhibiting the decline of 2B.

### Reopening the critical period by treating WT co-cultures with proBDNF

We also investigated whether the critical period once closed could be reopened by upregulating synaptic 2B expression in WT mice. ProBDNF enhances the NMDAR-dependent form of LTD and 2B-mediated synaptic currents by activating the p75 neurotrophin receptor (p75^NTR^)^26^. Conversely, deletion of p75^NTR^ impairs the NMDAR-dependent form of LTD and induces a substantial reduction in 2B, but not 2A, within the hippocampus^26^. This suggests proBDNF-p75^NTR^ controls 2B expression^26^. When we added 0.2 nM proBDNF to co-cultures, the amplitudes of 2B-CS-EPSCs more than doubled at 12–15 DIV (Fig. 1), and the levels of 2B within SPMs were also restored (Supplementary Fig. 2S). ProBDNF administrated after termination of the critical period reduced the amplitude of optical CS-EPSPs and the number of CS axons on the ventral side of the cord (Figs 2 and 3). These results indicate that the critical period can be reopened by restoring the levels of synaptic 2B to those seen during the critical period, which suggests closure of the critical period is governed by synaptic 2B.

### Prolongation of the critical period through partial blockade of inhibitory neural activity in the spinal cord

Manipulation of the excitatory-inhibitory input balance alters the onset and closure of the critical period in the developing visual cortex^21^. Inhibitory neural activity increases with development in the spinal cord^22^,^23^,^24^. Glycinergic inhibition predominates over GABAergic inhibition in the ventral spinal cord^27^, where the density of glycine receptors increases during development^22^,^23^. To test whether termination of the critical period can be affected by development of inhibitory neural activity in the spinal cord, we partially blocked glycinergic receptors by adding a low dose (0.2 μM) of strychnine to co-cultures for a period that extended beyond the end of the critical period (9–15 DIV)^28^,^29^. Electrophysiological recordings and immunoblot analyses showed that the strychnine had no effect on synaptic 2B levels (Supplementary Figs 3S and 4S), but it reduced the amplitudes of optical CS-EPSPs and the numbers of CS axons on the ventral side (Supplementary Figs 5S and 6S).

**Figure 4:**
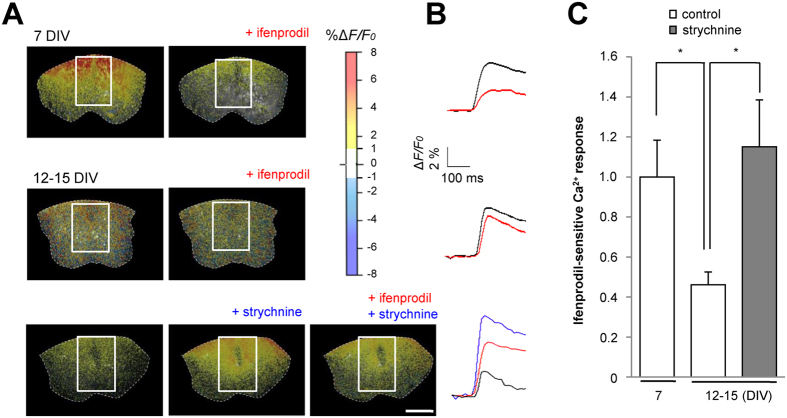
Ca^2+^ influx through 2B channels is enhanced by partial blockade of the inhibitory activity in the spinal cord. (**A**) Fluorescence imaging of Ca^2+^ using the Ca^2+^-sensitive dye fluo-4. Scale bar = 250 µm. (**B**) Time course of Ca^2+^ influx calculated from the fluorescence intensity at the indicated regions of interest. Calibration, Δ*F*/*F*_0_ 2%, 100 ms. (**C**) Ca^2+^ response (Δ*F*/*F*_0_) (1.00 ± 0.18 in control at 7 DIV, n = 23, Ns = 23, Nm = 14; 0.46 ± 0.06 in control at 12–15 DIV, n = 12, Ns = 12, Nm = 12; 1.15 ± 0.23 in strychnine, n = 18, Ns = 18, Nm = 16). *p < 0.05.

To evaluate the effect of reducing inhibitory neural activity on Ca^2+^ influx through 2B channels at CS synapses, the ifenprodil-sensitive portion of the Ca^2+^ signals was determined from the ratio of the fractional change in fluo-4 fluorescence before and after stimulation (Δ*F*/*F*_0_). Strychnine treatment increased Ca^2+^ entry through 2B channels to levels comparable to those seen during the critical period (Fig. 4). These results suggest that the development of inhibitory neural activity within the spinal cord contributes to ending the critical time window by reducing Ca^2+^ influx through 2B channels.

## Discussion

In the present study, electrophysiological and immunoblot analyses of an *in vitro* model system showed that synaptic expression of NMDAR subunit 2B decreased near the end of the critical period for activity-dependent corticospinal synapse elimination. *In vivo*, the developmental decline in synaptic 2B levels affects thalamic and cortical synapses^30^ and is accompanied by the replacement of subunit 2B with 2A^19^,^20^,^31^. Moreover, in the somatosensory and visual cortices, the critical periods end even in *2a*^−/−^ mice, where 2B cannot be replaced with 2A^32^,^33^. We showed here that in the *2a*^−/−^ spinal cord, levels of synaptic 2B are unchanged during the critical period and that blocking the decline of synaptic 2B prolongs the time window of plasticity. Further, upregulation of synaptic 2B levels in the WT spinal cord reactivated the plasticity time window after termination of the critical period. These data indicate that an increase in synaptic 2B levels is sufficient for the development of plasticity and suggest that the decline in synaptic 2B levels leads to closure of the critical period for CS plasticity.

It is technically possible to control the timing, duration and termination of the critical period in the developing visual system^21^. A brief reduction of GABAergic inhibition within the visual cortex reactivates the once-closed critical period for ocular dominance plasticity^34^. Thus the development of inhibitory circuitry appears to contribute to the closure of that critical period. In the present study, partial blockade of inhibitory activity of the spinal cord prolonged the critical period for CS plasticity. Under those conditions, although expression of synaptic 2B was unchanged, Ca^2+^ influx through 2B channels was significantly enhanced (Fig. 4).

NMDAR activation and the resultant increase in the postsynaptic Ca^2+^ concentration would be expected to alter the activity of downstream signaling cascades leading to the synapse elimination^35^,^36^,^37^,^38^. Evidence indicates that 2B is predominantly expressed early during development and is involved in various forms of developmental plasticity in the central nervous system^30^,^39^,^40^. It has also been suggested that some types of NMDAR-dependent plasticity, including the CS synapse elimination, depend on Ca^2+^ influx through 2B^16^,^25^,^41^ and that downstream signaling molecules specifically associated with 2B are responsible for this plasticity. This raises the possibility that developmental alteration in the activity of mediators downstream the Ca^2+^ influx determines the end of the critical period. In the present study, however, upregulation of synaptic 2B levels in the spinal cord was sufficient to reopen the critical plasticity window. This indicates that 2B, not its downstream signaling molecules, is the key determinant of critical period closure. Furthermore, the developmental increase in inhibitory neural activity within the spinal cord also plays an important role in determining the end of the critical period.

## Materials and Methods

All our experiments were approved by the Ethical Committee of Teikyo University School of Medicine and were performed in accordance with the Ethics Committee Guidelines for Animal Experimentation, Teikyo University School of Medicine (No. 14-009) and the National Institutes of Health Guide for the Care and Use of Laboratory Animals (NIH Publication No. 80-23) revised in 1996. All efforts were made to minimize the number of animals used and their suffering.

### Organotypic slice cultures

All experiments were performed using co-cultures of mouse sensorimotor cortex and cervical spinal cord slices as previously described^25^. Briefly, coronal cortical and axial spinal cord slices (350 μm thick) were cut starting on postnatal day 1 (P1) in WT and *2a*^−/−^ mice using a Linear slicer (Dosaka EM). The forelimb areas were then dissected from the cortical sections in cold cutting solution (120 mM choline-Cl, 3 mM KCl, 1.25 mM NaH_2_PO_4_, 28 mM NaHCO_3_, 8 mM MgCl_2_, and 25 mM glucose). The slices were placed on a collagen-coated membrane (Millicell, 0.4-μm pore, Merck Millipore) and maintained at 37 °C in an atmosphere containing 5% CO_2_. The genetic background of all mice was C57BL/6.

### Drug treatment

To block synapse elimination during the critical period, APV (50 μM) (TOCRIS) was added to the culture medium from 5 to 11 DIV. ProBDNF (0.2 nM) (Alomone Labs) was added to the medium from 13 to 16 DIV to increase synaptic expression of 2B after the critical period. Strychnine (0.2 μM) (Acros Organics) was used from 9 DIV to block inhibitory input in the spinal cord before the critical period ended. The drug-containing medium was then replaced with drug-free medium, and electrophysiological studies were performed 24 h later. The medium, including freshly prepared drugs, was changed twice weekly.

### Electrophysiological study

Slices from the culture insert, including the membrane, were placed in a recording chamber and superfused with artificial cerebrospinal fluid (ACSF) containing 119 mM NaCl, 2.5 mM KCl, 1 mM NaH_2_PO_4_, 26 mM NaHCO_3_, 1.29 mM MgSO_4_, and 2.24 mM CaCl_2_, saturated with 95% O_2_ and 5% CO_2_^24^. CS-EPSCs were evoked by applying current pulses (amplitude 500 μA, width 100 μs, 0.1 Hz) to the deeper layers of the cortical slices using a bipolar electrode. The internal solution in patch pipettes contained 128 mM Cs-gluconate, 20 mM CsCl, 10 mM HEPES-CsOH (pH 7.2–7.3), 0.2 mM EGTA, 2 mM ATP (Mg^2+^ salt) and 0.2 mM GTP (Na^+^ salt). Osmolarity was adjusted to 280 mOsm. NMDA currents were recorded in the presence of 10 μM CNQX or 5 μM NBQX and 50 μM picrotoxin in ACSF at a holding potential of +40 mV. Ifenprodil (3 μM, Sigma-Aldrich) or 10 μM Ro25-6981 (Sigma-Aldrich) was used to isolate 2B currents. Cells with a series resistance >30 MΩ or a leak current >100 pA were excluded from the analysis, and series resistance was compensated. Averaged amplitudes of the CS-EPSCs were measured using Clampfit (Axon Instruments).

### Optical recordings

A plasmid vector encoding hChR2 fused to EYFP (pAAV-CaMKIIa-hChR2 (H134R)-EYFP) was a kind gift from Dr. Deisseroth (Stanford University, Stanford, CA). Adeno-associated virus (AAV) encoding EYFP-tagged ChR2 (AAV-CaMKIIa-hChR2 (H134R)-EYFP) (AAV-ChR2-EYFP) was produced as previously described^12^, and the final particle titers were 2.6 × 10^10^ vector genomes (vg)/ml. We infected cortical slices with AAV-ChR2-EYFP by applying 1 μl of AAV (1.3 × 10^10 ^vg/ml) solution directly to cover only the surfaces of the cortical slices. The slices were then incubated for 2 h, washed with medium, and co-cultured with spinal cord slices. Cortices expressing ChR2-EYFP were illuminated using a 465-nm LED light for optical imaging. Because ChR2 is expressed along the entire surface of axon membranes, extending to their terminals, live imaging of ChR2-EYFP-labeled corticospinal axons was possible.

Slices were stained using the voltage-sensitive dye RH1691 (Optical Imaging), and optical measurements of changes of fluorescence were made using a high-speed camera system (MiCAM02, Brain Vision Inc.). Slices were incubated with the dye (1 mg/ml) for 20 min in an oxygen-saturated moist chamber and then placed in a perfusion chamber mounted on the stage of a fluorescence microscope (BX50WI, Olympus). After bubbling the stained slices with 100% O_2_ for 60 min, they were rinsed with ACSF (135 mM NaCl, 2.5 mM KCl, 2 mM CaCl_2_, 1.25 mM MgCl_2_, 10 mM HEPES, and 25 mM glucose, pH 7.35) and illuminated using a halogen lamp (excitation filter, 620–640 nm; absorption filter, *λ* > 665 nm). The acquisition time was 2.2 ms for each of the 1,028 frames chosen. Optical responses evoked by cortical illumination (465-nm LED light, 10 ms) were recorded. The intensity of RH1691 fluorescence at each pixel was divided by that of the brightest pixel in the image to obtain the normalized fluorescence, after which the percent fractional change in normalized fluorescence (%Δ*F*/*F*_0_) was calculated and designated as the optical signal. Data from pixels with a percent fractional change in normalized RH1691 fluorescence (%Δ*F/F*_*0*_) below the cut-off level (±0.1%) were excluded from the analysis. To show the spatial distribution of synaptic potentials in the spinal cord, we superimposed the pseudocolored optical image onto the *F*_*0*_image of the hemi-spinal cord. For quantitative measurements, two regions of interest were selected from dorsal and ventral areas of each hemi-spinal cord (Supplementary Fig. 1S). The ratios of the fractional changes in RH1691 fluorescence before and after stimulation (Δ*F*/*F*_0_) were calculated and designated optical CS-EPSPs.

### Calcium imaging

Slices were incubated with the membrane permeant AM ester of the Ca^2+^-sensitive dye fluo-4 (5 μg/ml; Molecular Probes) and 0.04% pluronic acid for 90 min at 37 °C, after which optical measurements of fluorescence changes were made using a high-speed camera system (MiCAM02). The slices were illuminated using a mercury lamp (100 W) equipped with a 470–495 nm excitation filter, and fluorescence was captured using a 510–550 nm absorption filter. The imaging area of the CCD camera was 8.4 × 6.5 mm^2^ comprising 376 × 252 pixels. The acquisition time was 10 ms for each of the 256 frames chosen. Optical responses evoked by cortical stimulation at 0.1 Hz were recorded. The ratios of the fractional changes in fluo-4 fluorescence to the fluorescence before stimulation (Δ*F*/*F*_0_) was calculated and used as the optical signal. Data from pixels with a %Δ*F/F*_*0*_ below the cut-off level (±1.0%) were excluded from the analysis. Nimodipine (20 μM) (Sigma-Aldrich) was applied to the medium to block Ca^2+^ influx through voltage-gated Ca^2+^ channels.

### Preparation of SPM fractions

Spinal cords were dissected from as many as 20 slice co-cultures per sample and were homogenized using a glass-teflon homogenizer in 10 volumes of ice-cold TEVP buffer containing 10 mM Tris pH 7.4, 5 mM NaF, 1 mM Na_3_VO_4_, 1 mM EDTA, 1 mM EGTA, 320 mM sucrose, and a protease inhibitor cocktail (Complete, Roche)^42^. The homogenate was then centrifuged at 1,000 × *g* for 10 min at 4 °C to remove the nuclear fraction (P1), after which the supernatant (S1) was centrifuged at 10,000 × *g* for 20 min at 4 °C to obtain the crude synaptosomal pellet (P2). This pellet was resuspended in 10 volumes of ice-cold TEVP buffer and centrifuged at 10,000 × *g* for 20 min at 4 °C to obtain the washed, crude synaptosomal fraction (P2′). That pellet was lysed in nine volumes of ice-cold hypo-osmotic solution containing 20 mM Tris pH 8.8, 5 mM EDTA, 1 mM Na_3_VO_4_ and protease inhibitor cocktail, and then homogenized using a glass-teflon homogenizer. After lysis for 30 min, the lysate was centrifuged at 25,000 × *g* for 30 min at 4 °C to obtain the SPM fraction (P3), which was suspended in SDS-PAGE sample buffer and stored at −20 °C.

### Antibodies

Rabbit polyclonal anti-NR2B (1:500 dilution) and mouse monoclonal anti-PSD-95 (clone 7E3-1B8; 1:500 dilution) antibodies were purchased from Millipore. A mouse monoclonal anti-pan-MAGUK scaffolding protein family antibody (clone K28/86, final concentration: 3.46 μg/ml) was purchased from the NIH NeuroMab Facility. This antibody was raised against a fusion protein composed of amino acid residues 77–229 of human PSD-95/SAP90 and cross-reacts with recombinant PSD-93, SAP97 and SAP102. ECL-anti-rabbit IgG, horseradish peroxidase-conjugated whole antibody (1:1000 dilution), and ECL-anti-mouse IgG, horseradish peroxidase-conjugated whole antibody (1:1000 dilution) were purchased from GE Healthcare.

### Immunoblot analysis

After sodium dodecyl sulfate-polyacrylamide gel electrophoresis (SDS-PAGE), the extracts of SPMs were electrophoretically transferred to polyvinylidene difluoride membranes (Immobilon-P membrane; Millipore) and probed with the indicated antibodies^43^. The immunoblots were developed using a chemiluminescence kit (SuperSignal West Femto Maximum Sensitivity Substrate, Thermo Scientific) and detected using an LAS3000 mini (Fujifilm). Scanned images were quantitated using Image Gauge software (Fujifilm). To quantify 2B within SPMs, the intensity of the 2B immunocomplex was normalized to that of immunocomplexes of MAGUK or PSD-95.

### Live imaging and enumeration of CS axons

For live imaging of fluorescently labeled CS axons in slice co-cultures, we employed an upright confocal microscope (FV1000, Olympus) equipped with a custom stage-top CO_2_ incubator system (Tokai Hit), which enabled acquisition of confocal images without damaging tissues^44^. Each slice co-culture was transferred to the stage-top CO_2_ incubator, and tiled images of up to nine microscope fields, including the entire spinal cord area, were captured using a 20× objective lens. We adjusted the brightness and contrast of the images using ImageJ 1.42q (Wayne Rasband, National Institutes of Health). To count the number of CS axons that crossed the 70% line drawn from the dorsal to the ventral edge of the spinal gray matter of the spinal cords in slice co-cultures, we used the ImageJ plugin “Find stack maxima”.

## Additional Information

**How to cite this article**: Isoo, N. *et al*. The decline in synaptic GluN2B and rise in inhibitory neurotransmission determine the end of a critical period. *Sci. Rep.* **6**, 34196; doi: 10.1038/srep34196 (2016).

## Supplementary Material

Supplementary Information
