# Supplementary material for: The decline in synaptic GluN2B and rise in inhibitory neurotransmission determine the end of a critical period
Source: Sci Rep. 2016 Sep 28;6:34196. doi: 10.1038/srep34196 (PMC5039748; doi:10.1038/srep34196)
Supplement: Supplementary Information [file srep34196-s1.pdf]

The decline in synaptic GluN2B and rise in inhibitory neurotransmission determine the end of a critical period

Noriko Isoo, Takae Ohno, Mutsumi Isowaki, Satoshi Fukuda, Naoyuki Murabe, Hiroaki Mizukami, Keiya Ozawa, Masayoshi Mishina, and Masaki Sakurai

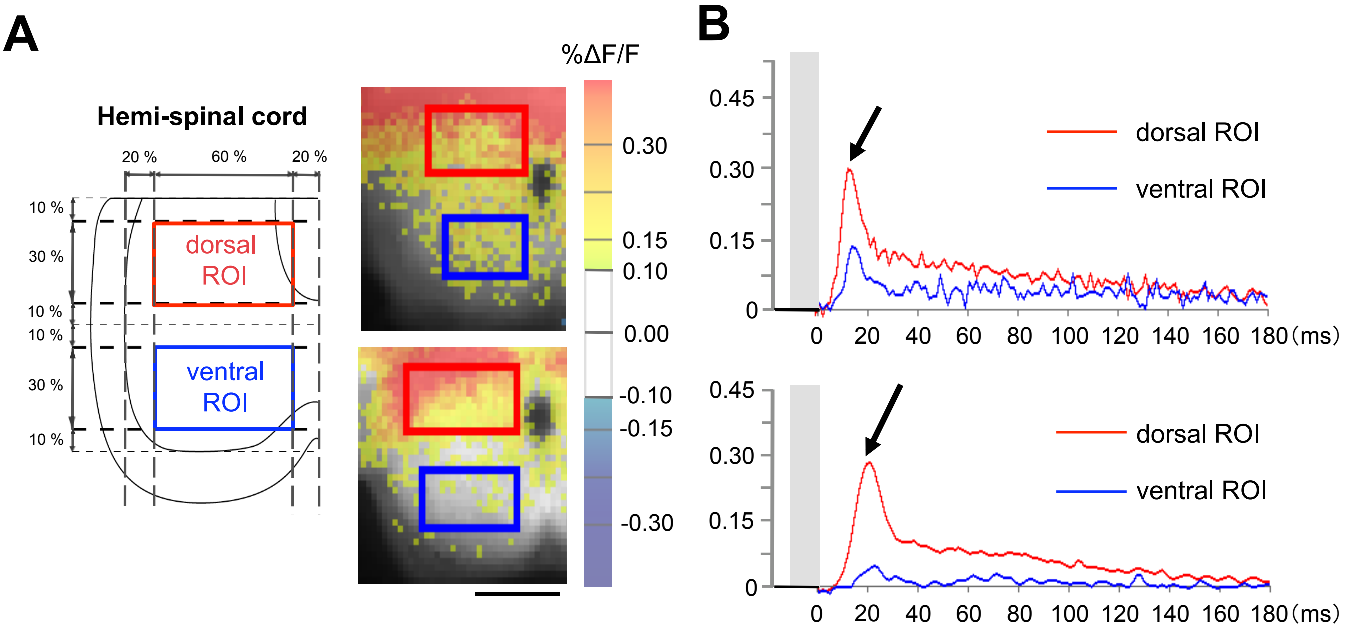

Supplementary Figure 1S Time course of optical CS-EPSPs recorded in dorsal and ventral ROIs in the spinal cord.
(A) ROIs in dorsal (red) and ventral (blue) hemi-spinal cord established for analyzing optical CS-EPSPs were shown. Scale bar = 250 μm. **(B)** The red and blue traces were optical signals recorded in the ROIs indicated on the images. ROI, regions of interest.

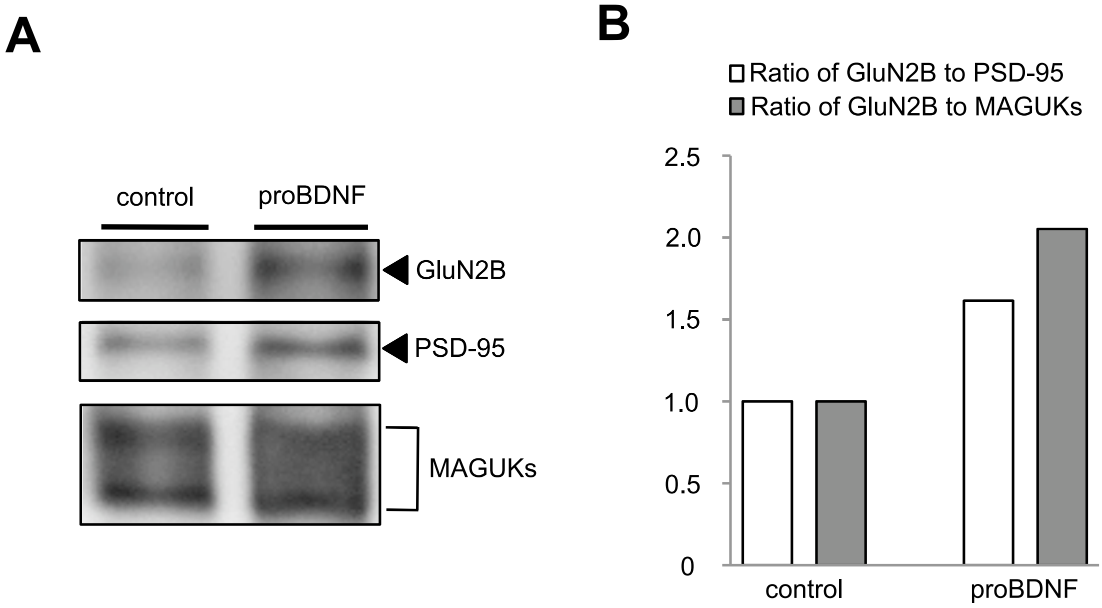

Supplementary Figure 2S Upregulation of synaptic 2B by proBDNF treatment.

(A) Immunoblot analysis of synaptic expression of 2B, PSD-95 and MAGUKs in control and proBDNF-treated spinal cords. **(B)** Ratios of 2B in SPMs to PSD-95 or MAGUK in SPMs in control or proBDNF-treated spinal explants at 16 DIV (Ratio of 2B for PSD-95: 1.00, control; 1.61, proBDNF-treated. Ratio of 2B for MAGUKs: 1.00, control; 2.05, proBDNF-treated. Each sample is an SPM extract prepared from 20 co-cultures from 4 mice).

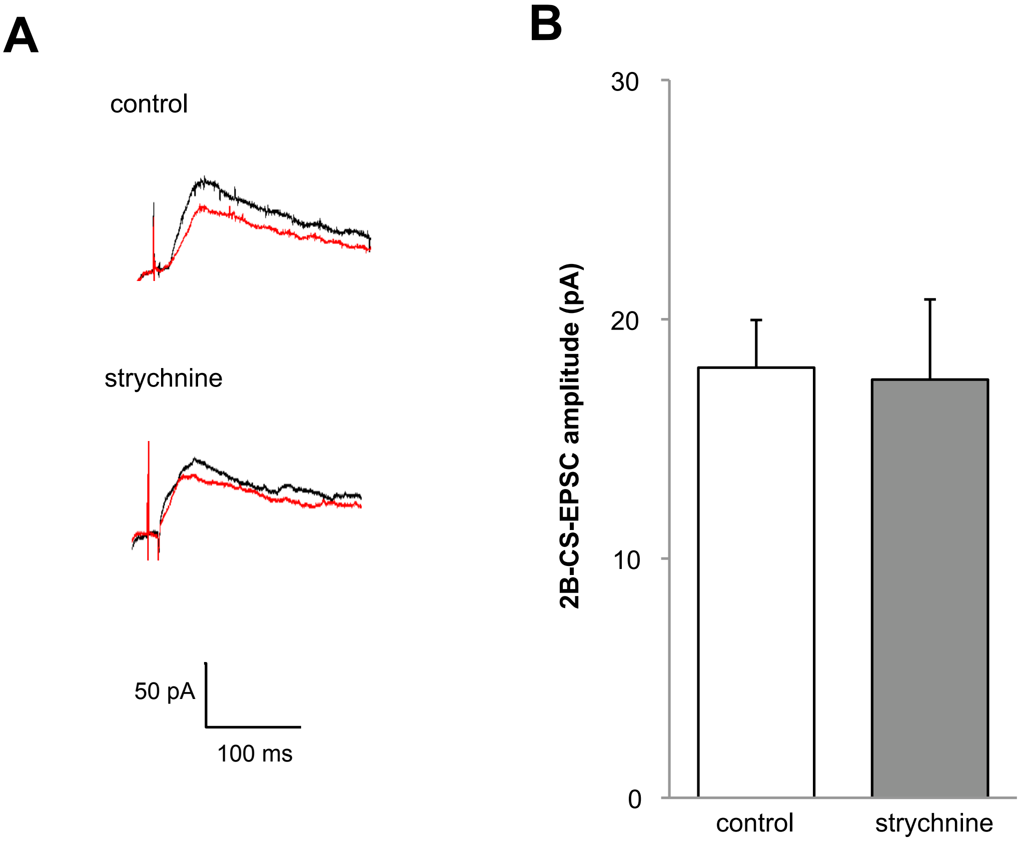

Supplementary Figure 3S No alterations in 2B-CS-EPSCs in control or strychnine-treated spinal explants.
(A) Averaged CS-EPSC traces recorded in control (top) or strychnine-treated (bottom) spinal explants before (black) or after (red) Ro25-6981 application at 12 DIV. **(B)** Averaged amplitudes of 2B-CS-EPSCs in control or strychnine-treated spinal explants at 12-15 DIV (control: n = 18, Ns = 10, Nm = 20; strychnine: n = 18, Ns = 12, Nm = 24). Calibration, 50 pA, 100 ms.

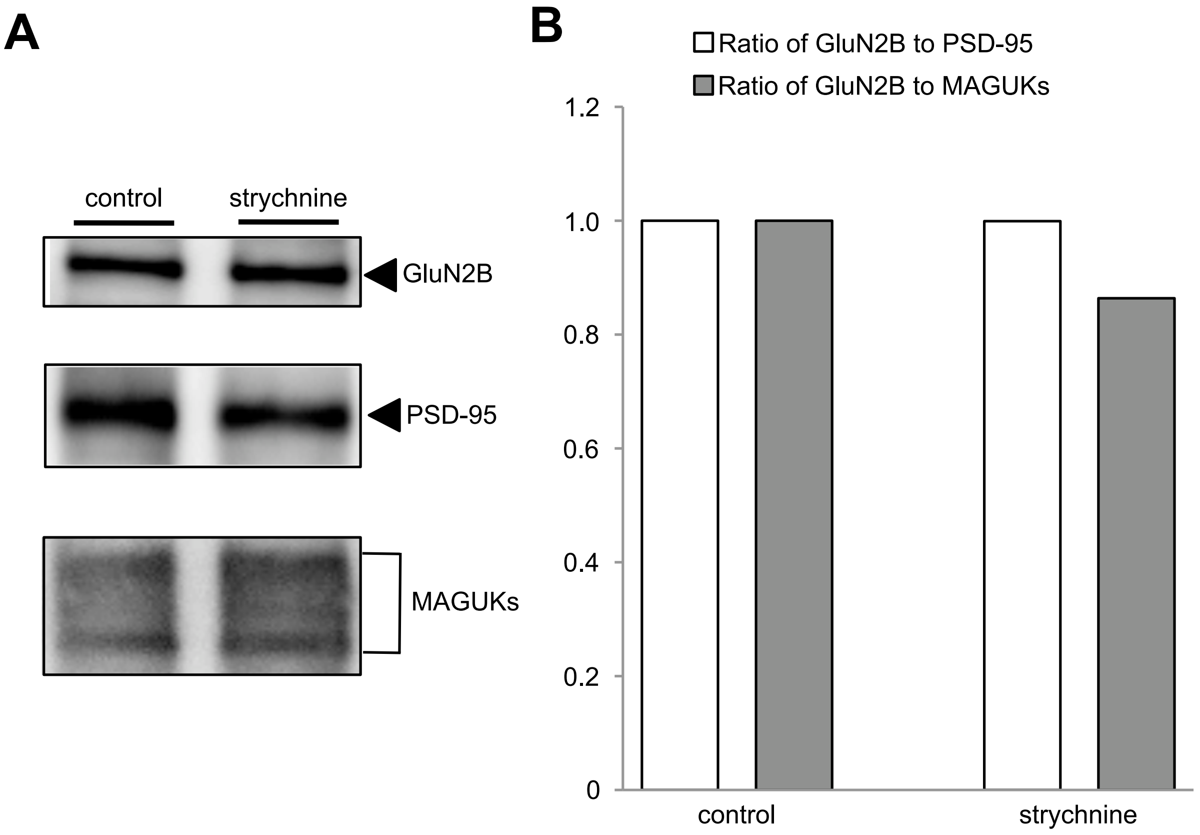

Supplementary Figure 4S Blockade of the inhibitory activity in the spinal cord.

(A) Immunoblot analysis of 2B, PSD-95 and MAGUKs in SPMs in control or strychnine-treated spinal cords. **(B)** Intensity of 2B in SPMs was normalized to that of PSD-95 or MAGUK in SPMs (ratio of 2B to PSD-95: 1.00 in control, 1.00 in strychnine-treated, ratio of 2B to MAGUKs: 1.00 in control, 0.86 in strychnine-treated, each sample is an SPM extract from 20 co-cultures from 4 mice).

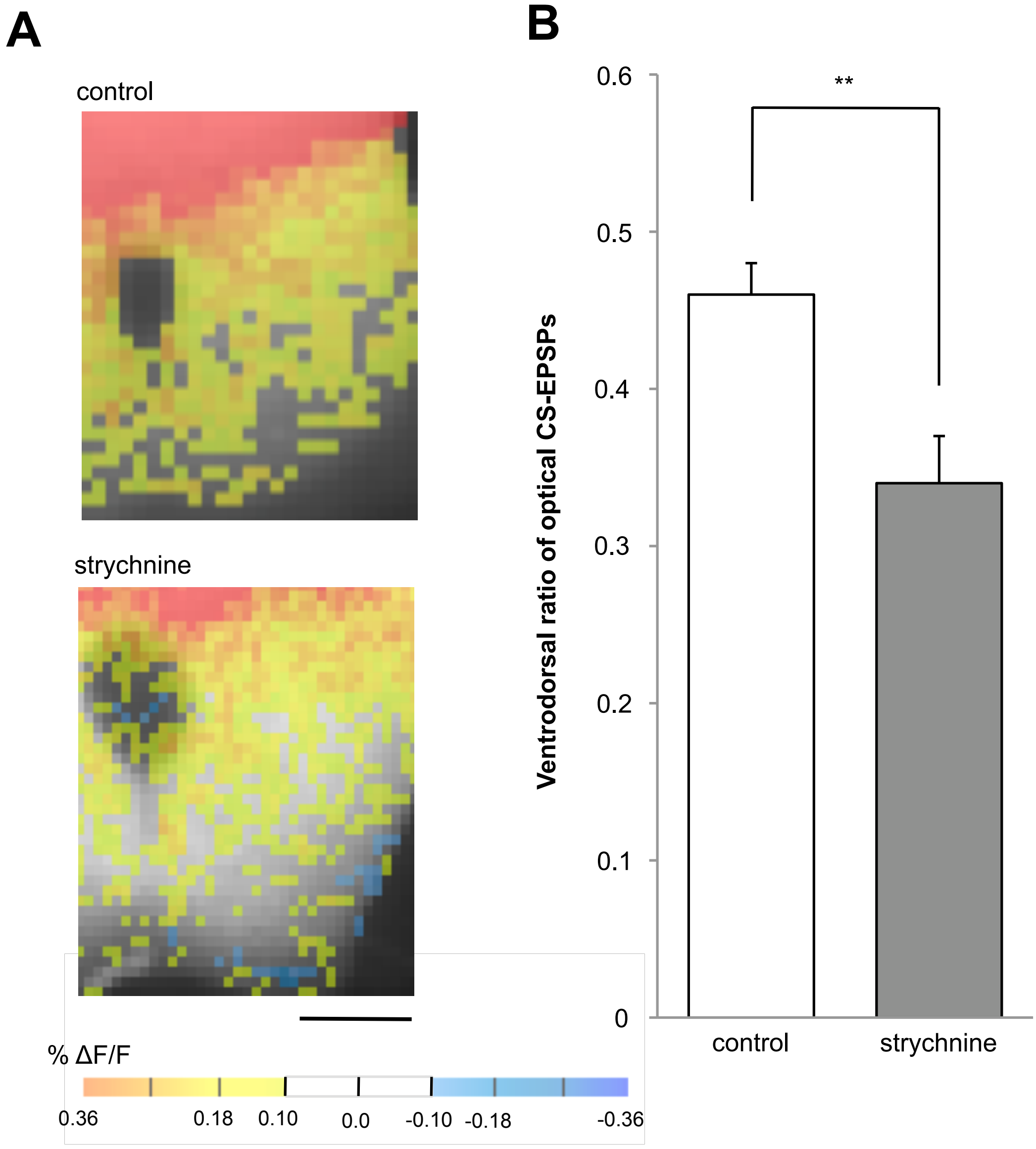

Supplementary Figure 5S CS synapses in the ventral spinal cord were eliminated by strychnine treatment.

(A) Spatial distribution of CS synapses determined using optical CS-EPSPs in control (top) or strychnine-treated (bottom) spinal explants. Scale bar = 250 μm.

(B) Ventrodorsal ratios of optical CS-EPSPs in control (0.46 ± 0.02, n = 17, Ns = 17, Nm = 8) and strychnine-treated (0.34 ± 0.03, n = 8, Ns = 8, Nm = 6) spinal explants at 14-16 DIV.

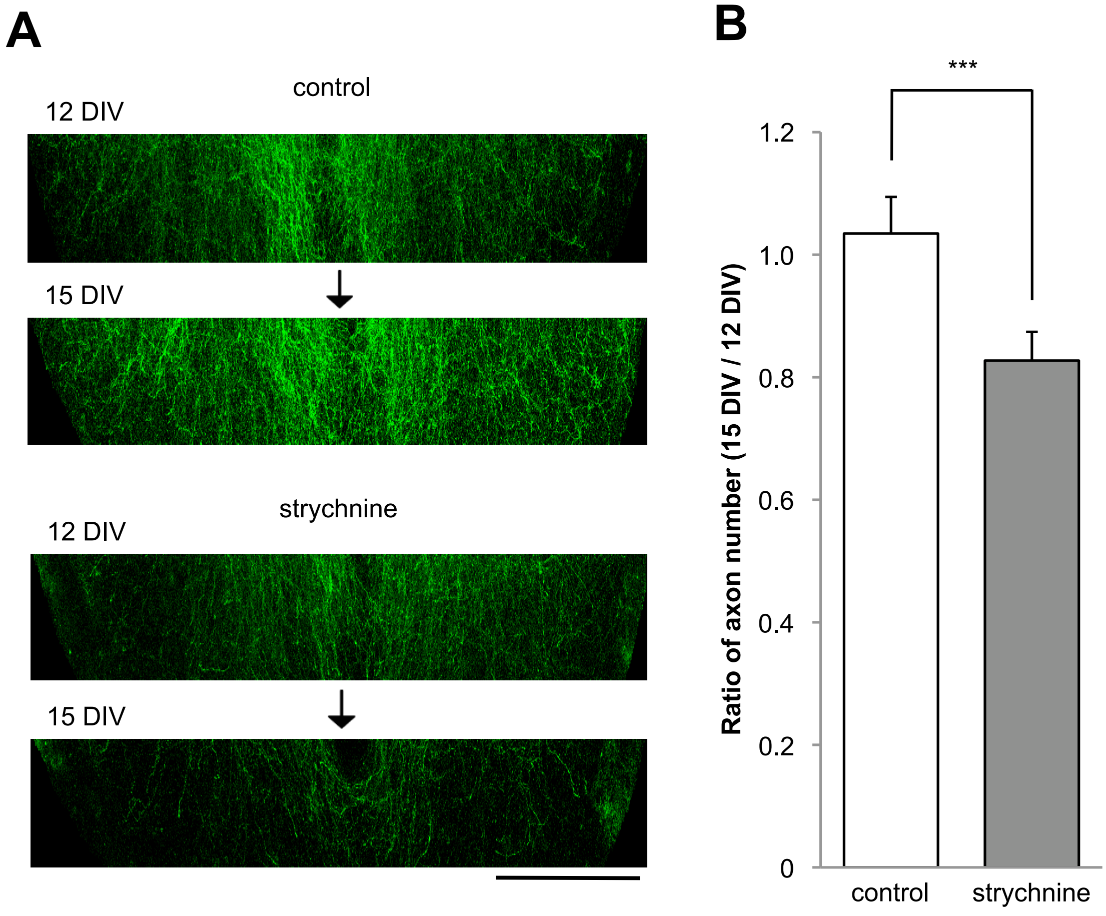

Supplementary Figure 6S Strychnine elicited regression of CS axons on the ventral side of the spinal cord.

(A) Images of CS axons on the ventral side that crossed the 70% line from the dorsal to the ventral edge of spinal gray matter in control (top) and strychnine-treated (bottom) spinal explants. The first and second images were taken at 12 and 15 DIV, respectively. **(B)** Ratio of the number of CS axons at 15 DIV to that at 12 DIV (1.03 ± 0.06 in control, n = 9, Ns = 9, Nm = 4; 0.83 ± 0.05 in strychnine-treated, n = 10, Ns = 10, Nm = 4).

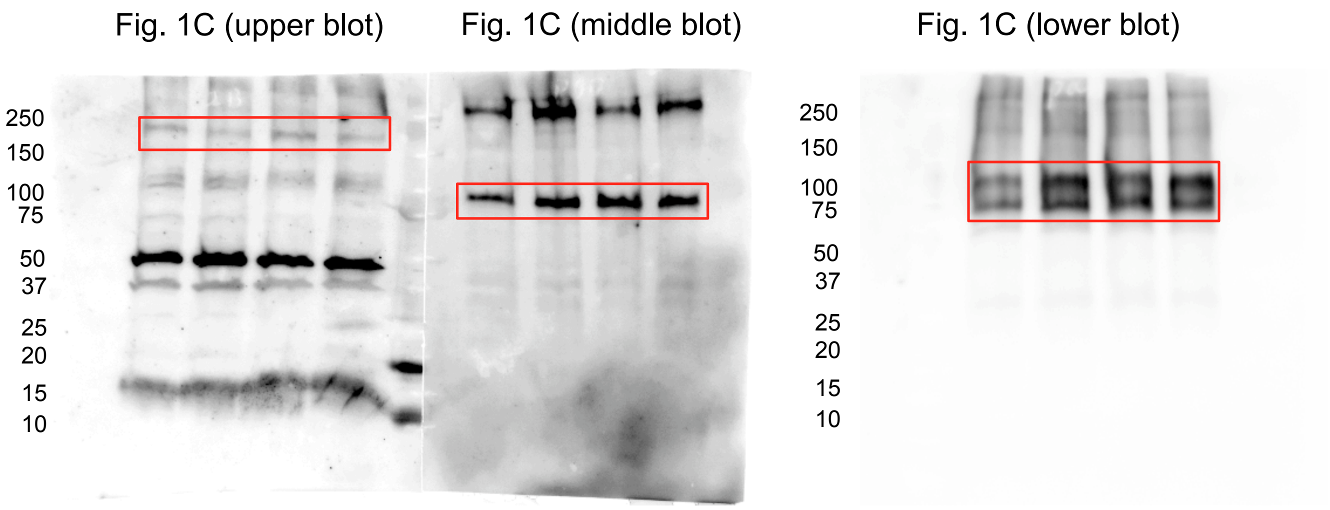

Supplementary Figure 7S Uncropped western blots.
The red boxes indicate the cropped regions.
